# The Repellent DEET Potentiates Carbamate Effects *via* Insect Muscarinic Receptor Interactions: An Alternative Strategy to Control Insect Vector-Borne Diseases

**DOI:** 10.1371/journal.pone.0126406

**Published:** 2015-05-11

**Authors:** Aly Abd-Ella, Maria Stankiewicz, Karolina Mikulska, Wieslaw Nowak, Cédric Pennetier, Mathilde Goulu, Carole Fruchart-Gaillard, Patricia Licznar, Véronique Apaire-Marchais, Olivier List, Vincent Corbel, Denis Servent, Bruno Lapied

**Affiliations:** 1 Laboratoire Récepteurs et Canaux Ioniques Membranaires (RCIM) UPRES EA 2647/USC INRA 1330, SFR 4207 QUASAV, Université d’Angers, UFR SCIENCES, Angers cedex, France; 2 Faculty of Biology and Environment Protection, N. Copernicus University, Torun, Poland; 3 Institute of Physics, Faculty of Physics, Astronomy and Informatics, N. Copernicus University, Torun, Poland; 4 Institut de Recherche pour le Développement, UMR 224 Maladies Infectieuses et Vecteurs: Ecologie, Génétique, Evolution et Contrôle (MiVEGEC), Montpellier, France; 5 CEA, iBiTecS, Service d’Ingénierie Moléculaire des Protéines (SIMOPRO), Laboratoire de Toxinologie Moléculaire et Biotechnologie, Gif sur Yvette, France; 6 Department of Entomology, Faculty of Agriculture at Kamphaeng Saen, Kamphaeng Saen Campus, Kasetsart University, Nakhon Pathom, Thailand; 7 Plant Protection Department, Faculty of Agriculture, Assiut University, Assiut, Egypt; Weizmann Institute of Science, ISRAEL

## Abstract

Insect vector-borne diseases remain one of the principal causes of human mortality. In addition to conventional measures of insect control, repellents continue to be the mainstay for personal protection. Because of the increasing pyrethroid-resistant mosquito populations, alternative strategies to reconstitute pyrethroid repellency and knock-down effects have been proposed by mixing the repellent DEET (N,N-Diethyl-3-methylbenzamide) with non-pyrethroid insecticide to better control resistant insect vector-borne diseases. By using electrophysiological, biochemichal, *in vivo* toxicological techniques together with calcium imaging, binding studies and *in silico* docking, we have shown that DEET, at low concentrations, interacts with high affinity with insect M1/M3 mAChR allosteric site potentiating agonist effects on mAChRs coupled to phospholipase C second messenger pathway. This increases the anticholinesterase activity of the carbamate propoxur through calcium-dependent regulation of acetylcholinesterase. At high concentrations, DEET interacts with low affinity on distinct M1/M3 mAChR site, counteracting the potentiation. Similar dose-dependent dual effects of DEET have also been observed at synaptic mAChR level. Additionally, binding and *in silico* docking studies performed on human M1 and M3 mAChR subtypes indicate that DEET only displays a low affinity antagonist profile on these M1/M3 mAChRs. These results reveal a selective high affinity positive allosteric site for DEET in insect mAChRs. Finally, bioassays conducted on *Aedes aegypti* confirm the synergistic interaction between DEET and propoxur observed *in vitro*, resulting in a higher mortality of mosquitoes. Our findings reveal an unusual allosterically potentiating action of the repellent DEET, which involves a selective site in insect. These results open exciting research areas in public health particularly in the control of the pyrethroid-resistant insect-vector borne diseases. Mixing low doses of DEET and a non-pyrethroid insecticide will lead to improvement in the efficiency treatments thus reducing both the concentration of active ingredients and side effects for non-target organisms. The discovery of this insect specific site may pave the way for the development of new strategies essential in the management of chemical use against resistant mosquitoes.

## Introduction

In recent years, because of the increasing pyrethroid-resistant mosquito populations [1,2], repellents and particularly DEET, considered as the standard product against mosquitoes, have gained increasing interest in public health for protecting people. Previous data have indicated that DEET displays a complex broad-spectrum action. It affects various types of insect sensory receptor neurons [3–12] such as olfactory receptor neurons, odorant receptors, and gustatory receptor neurons conditioning insect avoidance behavior and it alters fine-tuning of functionally olfactory receptor neurons. Additionally, it has been reported that DEET is not only a behaviour-modifying agent. It blocks, at peripheral nervous system level, insect neuromuscular junction and affects central octopaminergic synapses to induce neuroexcitation and toxicity [13]. Furthermore, DEET is also considered as a reversible inhibitor of insect acetylcholinesterase (AChE), an enzyme involved in the rapid hydrolysis of the neurotransmitter acetylcholine at cholinergic synapses in the central nervous system and is able to strengthen the toxicity of anticholinesterase insecticides such as carbamates [14]. According to these data, alternative strategies to reconstitute pyrethroid repellency and knock-down effects have been proposed by mixing DEET with non-pyrethroid insecticide to better control resistant insect vector-borne diseases [15–17]. However, except few findings, which report that cytochrome-P450 monooxygenases are responsible for the enhanced toxicity observed between DEET and the carbamate propoxur in mosquitoes *Aedes aegypti* [18], the underlying cellular mechanisms involved in the synergism remain unknown. In the cockroaches *Periplaneta americana* central nervous system, pacemaker neurosecretory cells, named the Dorsal Unpaired Median (DUM) neurons [19,20] are known neuronal model used for electro-pharmacological studies [21]. Because adult DUM neuron cell bodies express different cholinergic receptors including nAChR resistant to α-bungarotoxin, muscarinic AChR subtypes (mAChR) and AChR with “mixed” nicotinic-muscarinic pharmacology activated by ACh, which is regulated by AChE [22–24], they represent a suitable cellular model to investigate the mode of action of both repellents and insecticides on the insect cholinergic system. Consequently, the following study has been designed to bring new insights on the neurophysiological action of DEET and to identify new molecular targets underlying the synergistic effect.

## Results and Discussion

### Positive interaction between DEET and propoxur

**Fig 1:**
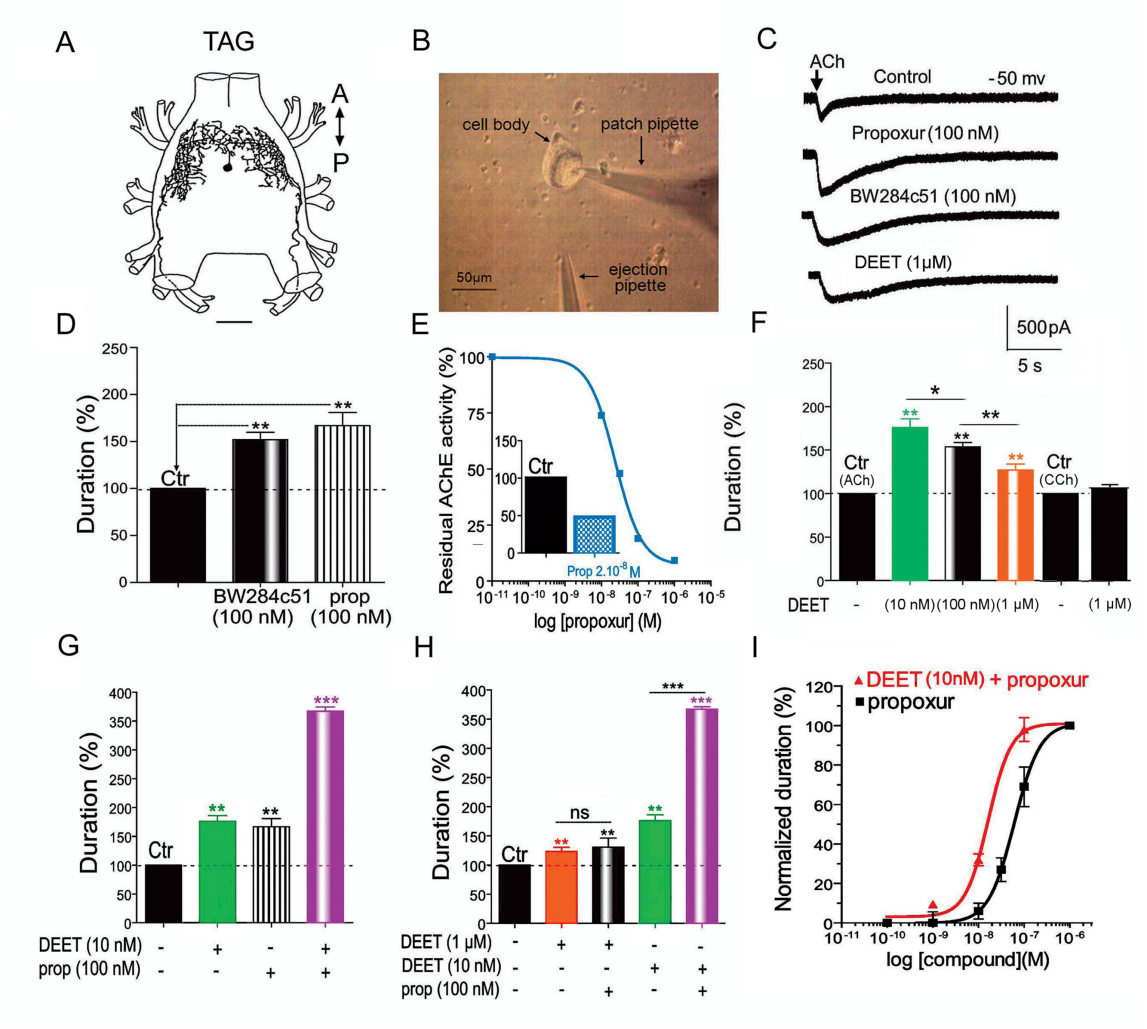
DEET potentiates the carbamate-induced anticholinesterase effect in insect DUM neurons. A) Dorsal view *camera lucida* drawing of typical DUM neuron morphology revealed by anterograde cobalt staining performed on a soma located along the midline of the cockroach terminal abdominal ganglion (TAG) of the nerve cord. A, anterior; P, posterior; scale bar 120μm. B) Light micrograph of the whole cell patch-clamp technique adapted on the isolated DUM neuron cell body obtained after enzymatic digestion and mechanical dissociation of the TAG. C) Anticholinesterase effects of the carbamate, propoxur, the anticholinesterase compound BW284c51 and the repellent DEET on the duration of the ACh-induced inward currents (measured at 50% of the maximum current amplitudes) obtained in whole-cell voltage-clamp at a steady-state holding potential of -50 mV. D) Comparative bar graph summarizing the anticholinesterase effect of the specific inhibitor BW284c51 (100nM) and the carbamate, propoxur (prop) (100nM) measured on the duration of the ACh-induced inward currents (measured at 50% of the maximum current amplitudes) obtained in whole-cell voltage-clamp at a steady-state holding potential of -50 mV. E) Concentration-dependent inhibition of the residual AChE activity determined spectrophotometrically induced by propoxur and expressed as percentage of initial activity (i. e., without propoxur). The curve represents the best fit to the data points according to the Hill equation yielding the corresponding IC_50_ (i.e., the concentration of propoxur that produces 50% inhibition of the AChE enzymatic activity) as illustrated in the comparative bar graph shown *in inset*. This indicates that isolated DUM neurons express functional AChE. F) Bar graph summarizing the unexpected concentration-dependent effect of DEET on the ACh-induced inward current duration. At low concentration (10nM), DEET produces a more important anticholinesterase effect than those observed with higher concentrations (i.e., 100nM and 1μM). By contrast, DEET (1μM) do not produce any effect on the carbachol(CCh)-induced current. G) Comparative bar graph illustrating the anticholinesterase effects of DEET (10nM) and propoxur (100nM) tested alone and in combination (DEET/propoxur). Pretreatment of DUM neuron with low concentration of DEET (10nM), for 15 minutes, strongly potentiates the propoxur-induced anticholinesterase effect. H) Comparative bar graph showing that synergistic effect between DEET and propoxur is only observed at low concentration of DEET (i. e., 10nM) and not with higher concentration (i. e., 1μM). I) Semi-logarithmic concentration-response curves for the anticholinesterase effect induced by propoxur applied alone and in the presence of 10nM DEET. The sigmoid curves represent the best fit to the mean data points according to the Hill equation yielding the corresponding IC_50_ of 2.10^-8^M and 6.10^-8^M estimated for DEET and propoxur applied in combination and for propoxur applied alone, respectively. Number of experiments varies from 10 to 16 cells. Data are means ± S.E.M. ** and ***, values significantly different, *p* < 0.01 and *p*<0.001, respectively; ns, not significant (*p* > 0.05).

DUM neuron cell bodies (Fig 1A and 1B) display membrane potential properties regulated by the activation of cholinergic receptors [22–24] (S1A Fig). We first demonstrated the contribution of AChE in modulating the ACh response in DUM neurons with the selective AChE inhibitor, BW284c51 [25] (100nM) and propoxur (100nM), an anticholinesterase carbamate insecticide. In both cases, the toxic activity resulted in an increase of the duration of the ACh-induced current (measured at 50% of the full current amplitude) obtained at a steady-state holding potential of -50 mV, following pneumatic pressure application of ACh (Fig 1B–1D). This indicates that inhibition of ACh hydrolysis prolongs the effect of ACh. Expression of AChE in DUM neurons was confirmed biochemically by measuring AChE enzymatic activity in the presence of propoxur. The carbamate inhibited, in a concentration-dependent manner, the AChE enzymatic activity yielding an IC_50_ value of 2.10^-8^M (Fig 1E). These results reveal that isolated DUM neuron cell bodies express membrane-bound AChE. Electrophysiological experiments performed with the repellent DEET (1μM), indicated that it produced an anticholinesterase effect, as previously observed at synaptic level [14]. It should be indicated that the current mediated by carbamylcholine (CCh), a non-hydrolysable cholinergic agonist was not modified in the presence of 1μM DEET (Fig 1F). The anticholinesterase effect of DEET occurred in an unusual concentration-dependent manner. Very low concentration (10nM) produced a stronger effect on the ACh-induced current duration than that of induced by 1μM (Fig 1F). Furthermore, pre-treatment with DEET (1μM) or propoxur (100nM), applied alone prevented any additional anticholinesterase effects of DEET/propoxur mixture (S1B and S1C Fig). Interestingly, co-application of lower concentration of DEET (10nM) with propoxur (100nM) on DUM neurons pre-treated with DEET (10nM) resulted in a strong synergistic anticholinesterase effect of propoxur (Fig 1G) never observed with higher concentration of DEET (1μM; Fig 1H). Propoxur effects alone and in the presence of 10nM DEET were also measured more quantitatively in DUM neurons. Mean values for percentage of ACh-induced current durations were plotted against the logarithm of the non-cumulative concentration of propoxur. The sigmoid curve, corresponding to the best fit (correlation coefficient r = 0.998) according to the Hill equation, gave an IC_50_ value for propoxur alone of 6.10^-8^M (Fig 1I). In the presence of DEET (10nM), we observed a significant shift to the left in a parallel manner of the curve of propoxur (IC_50_ DEET/propoxur 2.10^-8^M; Fig 1I). Pre-treatment with low concentration of DEET renders AChE more sensitive to the carbamate propoxur.

**Fig 2:**
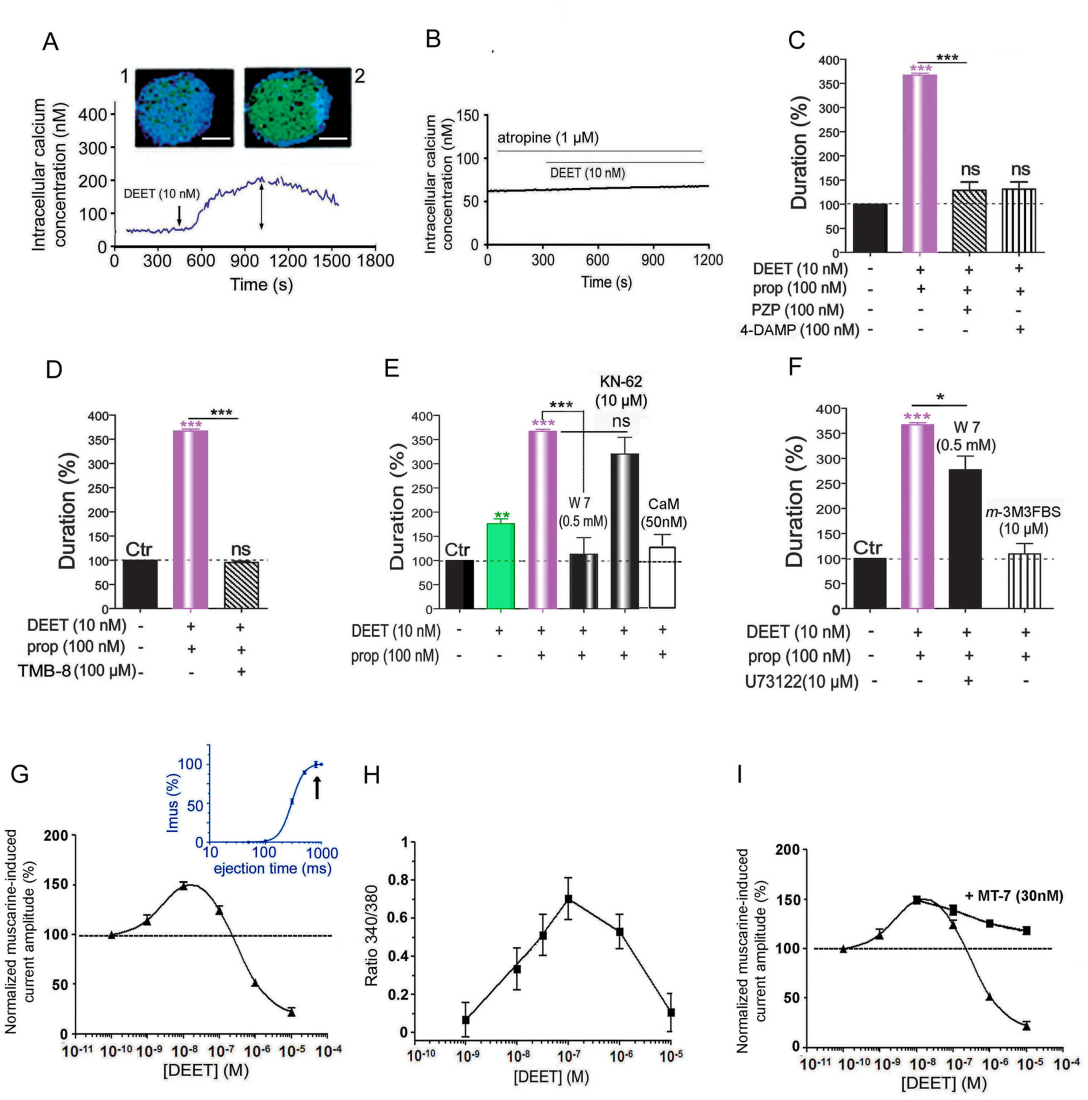
Synergism between DEET and propoxur occurs through a positive allosteric-like modulation of insect M1/M3 muscarinic receptors and intracellular calcium-dependent signaling pathways. A) Bath application of 10nM DEET increases intracellular free calcium concentration ([Ca^2+^]_i_) in Fura-2 loaded DUM neurons (*inset* 2). Note that, under control condition, calcium spark-like events are detected (*inset* 1). B) Pretreatment with 1μM atropine, a specific antagonist of muscarinic receptors (mAChRs), completely blocks the enhancement of [Ca^2+^]_i_ produced by 10nM DEET, indicating the involvement of mAChRs. C) Bar graph summarizing the inhibitory effect of M1 and M3 mAChR antagonists pirenzepine (PZP) and 4-DAMP, respectively, on the synergism between DEET and propoxur. D) Bar graph illustrating that TMB-8 (100μM) also completely blocks the synergism between DEET and propoxur. E,F) Characterization of the intracellular calcium-dependent molecular events involved in the synergistic action of DEET on the propoxur-induced anticholinesterase effect. Intracellular application of 0.5mM W7, the calmodulin inhibitor and 50nM calmodulin (CaM) inhibit the positive potentiating effect of DEET on the toxic activity of propoxur. By contrast, KN-62 (10μM), which binds to CaM kinase II and blocks its activation by calmodulin, does not produce any effect (E). If pretreatment with 10μM of U73122, an inhibitor of PI-PLC known to regulate AChE activity, partially counteracts the effect of 0.5mM W7, application of the PI-PLC activator, *m*-3M3FBS (10μM) produces similar inhibition of the synergism between DEET and propoxur to that of observed with W7 tested alone (F). G) Modulation of the maximum amplitude of muscarine-elicited currents *versus* the concentration of DEET applied. The limited window of DEET concentration within which a maximum response potentiating effect is observed, is around 10nM (G). For higher DEET concentrations, the sensitizing effect is counteracted and eventually outweighed by an inhibitory action of DEET. Inset illustrates the semi-logarithmic dose-response curve for the muscarine-induced current applied by pressure ejection. Arrow indicates that the maximum current amplitude is obtained for pressure ejection duration of 500ms. H) DEET induces a transient concentration-dependent [Ca^2+^]i rise in Fura-2-loaded DUM neuron cell bodies. The changes in [Ca^2+^]_i_ response amplitudes and the window of concentrations for DEET action were very similar with those illustrated in 2G. **(I)** Bath application of MT-7 (30nM), which is known to bind on M1 mAChR allosteric site, partially reversed the inhibitory effect observed for high concentration of DEET. Number of experiments varies from 8 to 13 cells. Data are means ± S.E.M. *, ** and ***, values significantly different, *p* < 0.05, *p* < 0.01 and *p* < 0.001, respectively. ns, not significant (*p* > 0.05). Scale bar: 20μm.

It is known that calcium-dependent phosphorylation/dephosphorylation process modulates sensitivity of targets to insecticides [26–30]. To determine whether the DEET-induced potentiation effect was mediated through calcium mobilization, calcium imaging experiments were performed on Fura2-loaded DUM neurons (Fig 2A). Bath application of DEET (10nM) produced a relatively slow elevation of intracellular calcium concentration ([Ca^2+^]_i_). The cytofluorescence intensity was first detected in the middle part of the cell whereas there was no fluorescence detected at the cell body periphery (*inset* 2; Fig 2A). This illustrates that calcium rise mostly involved calcium from intracellular stores. Because i) experiments were performed in the presence of α-bungarotoxin to inhibit “mixed” AChR and ii) DUM neuron nAChRs are not permeable to calcium [22–24], the involvement of muscarinic acetylcholine receptor (mAChR) was suspected. This was confirmed by using atropine (1μM), a specific mAChR antagonist, which completely blocked the calcium rise induced by 10nM DEET (Fig 2B). It should be mentioned that high extracellular potassium concentration, known to depolarize DUM neurons, still increased [Ca^2+^]_i_ in the presence of 1μM atropine (not shown). It is also interesting to mention that calcium spark-like events were detected in isolated DUM neuron under control conditions (*inset* 1; Fig 2A). These events, which completely disappeared in the presence of 1μM atropine suggest that DUM neurons display active ACh-activated mAChRs present in the membrane vicinity that contribute to regulate electrical activity. The use of more selective M1/M3 mAChR antagonists, pirenzepine (100nM) and 4-DAMP (100nM) inhibited the synergy between DEET and propoxur (Fig 2C). Furthermore, the antagonists only reduced the effects of DEET on the ACh-induced current duration (10nM) but not those induced by propoxur (S2A and S2B Fig), suggesting a possible effect of DEET *via* mAChRs.

### Synergism between DEET and propoxur occurs *via* mAChRs

M1/M3 mAChRs are known to be mainly coupled to phospholipase C (PLC) second messenger pathway. To examine if the synergistic effect of DEET was related to the downstream activation of inositol triphosphate (IP3) receptors which thereby increase [Ca^2+^]i, TMB-8 (100μM), an IP3 receptor antagonist, was tested. TMB-8 inhibited the DEET-induced potentiating effect (Fig 2D), confirming the role of calcium released from internal stores. The influence of [Ca^2+^]i was confirmed with caffeine, known to stimulate the release of calcium from internal stores and BAPTA, a fast efficient calcium chelator. If caffeine (10mM) mimicked the effect of DEET by increasing the anticholinesterase action of propoxur, BAPTA (10mM) decreased the effect of carbamate (S2C Fig). These results together with those relating that DEET, even in the presence of pirenzepine, potentiate the effect of propoxur when relatively high calcium was introduced into the DUM neuron through the patch pipette (no EGTA, S2D Fig) argue in favor of the regulatory role of intracellular calcium on AChE sensitivity, modulating the potentiated effect of propoxur.

We next focused our study on the characterization of the calcium-dependent events involved in the regulation of AChE sensitivity to propoxur, following changes in [Ca^2+^]_i_ induced by low concentration of DEET. In insect, calcium acting through the calcium-receptor protein calmodulin (CaM) is an important signal that regulates diverse enzymatic activities [24]. The possible regulatory role of the calcium/CaM complex in the AChE sensitivity to propoxur was examined using the calmodulin inhibitor W7 (0.5mM). W7 completely suppressed the potentiation previously observed (Fig 2E). KN-62 (10μM), known to block CaM-kinase II activated by the calcium/CaM complex in DUM neurons [26,27,29] did not produce any effect on the DEET-induced potentiation (Fig 2E). Thus, it seems that calcium/CaM complex indirectly regulates AChE sensitivity. This hypothesis is reinforced by recent knowledge about the molecular architecture of AChE [31]. AChEs exist in multiple molecular forms reflecting differences in their mode of attachment to cellular membranes. In insects, the mode of attachment is a glycophosphatidylinositol (GPI) anchor. This GPI anchor, covalently attached to the hydrophobic domain of the AChE C-terminus, could be sensitive to the action of phosphatidylinositol specific phospholipase C (PI-PLC) [32], an enzyme which is regulated by a calcium-dependent mechanism. Additional experiments were carried out with U73122 and *m*-3M3FBS, the specific inhibitor and activator of phospholipase C, respectively [33]. Application of 10μM *m*-3M3FBS inhibited the synergy between DEET and propoxur (Fig 2F). Moreover, in the presence of 10μM U73122, it was possible to counteract the inhibition produced by intracellular application of W7 (0.5mM), which blocked the synergism (Fig 2E and 2F). This indicates that DEET-induced intracellular calcium rise regulates negatively PI-PLC through the calcium/CaM complex formation rendering AChE more sensitive to propoxur. By contrast, the absence of significant intracellular calcium elevation observed with high concentration of DEET (e.g., Fig 2H) could result in a constitutive positive modulation of PI-PLC by CaM alone, inducing a less sensitive AChE to the carbamate. This was confirmed by applying intracellularly excess calmodulin (50nM), which strongly reduced the synergism (Fig 2E). This opposite dual effect depending on the concentration, leads us to consider DEET as a positive and/or negative modulator for insect M1/M3 mAChRs, depending on the concentration used.

To test this hypothesis, we performed experiments using pressure ejection application of muscarine. The average muscarine-induced inward current amplitudes were plotted *versus* different concentrations of DEET. The biphasic concentration-response curve (Fig 2G) indicated that the sensitizing effect of DEET on the muscarinic response was produced in a limited window of concentrations, which began at 10^-9^M and extended to about 10^-8^M. In this case, the DEET-induced potentiation of the current amplitude was not due to a direct effect on M1/M3 mAChR orthosteric site since muscarine applied by 500ms pressure ejection in duration resulted in full saturation of the receptors (*inset* Fig 2G). Beginning after 10^-8^M, the sensitizing effect was counteracted and outweighed by an inhibitory action of DEET. Interestingly, similar bell-shaped relationship was observed by measuring M1/M3 mAChR-induced [Ca^2+^]_i_ variations in the presence of different concentrations of DEET (Fig 2H). From these results, we then propose that DEET, at low concentrations, may act as a positive allosteric modulator of the muscarine action on mAChRs. Because DEET used at high concentrations exerted an inhibitory action (Fig 2G and 2H), we postulated that DEET may interact with a distinct low affinity site on M1/M3 mAChR.

We then examined the effect of the Muscarinic Toxin 7 (MT-7), known to bind specifically with the allosteric site of the M1 mAChR [34]. Pretreatment of isolated DUM neuron with 30nM MT-7 counteracted the effect of DEET only for concentrations higher than 10^-8^M, corresponding to the inhibitory action of the repellent on M1/M3 mAChRs (Fig 2I). MT-7 interaction blocks selectively the accessibility of DEET to its low affinity interaction site without affecting its binding to the high affinity site. Taken together, our results suggest that DEET, at low concentrations, induces a positive allosteric effect of muscarinic agonist function on insect M1/M3 mAChRs. This produces [Ca^2+^]_i_ rise and the calcium/CaM complex formation. At high concentrations, DEET produces opposite effects by interacting with a low affinity site. This unusual concentration-dependent dual opposite effects of DEET was also observed, at synaptic level, on postsynaptic mAChRs known to be involved in the modulation of integrative properties of the giant interneurons [35]. Using the single oil-gap technique (S3A Fig), it is possible to record unitary excitatory postsynaptic potential (S3B Fig) resulting from the activity of presynaptic cercal mechanoreceptors [36] and to measure the postsynaptic membrane potential [35,36] (S3C Fig). Bath application of low concentrations of DEET (100nM and 500nM) produced a concentration-dependent slow postsynaptic membrane depolarization completely blocked by 1μM atropine, whereas higher concentrations (10μM), produced an inhibitory action (S3C and S3D Fig).

### Synergy between DEET and propoxur against *Ae*. *Aegypti*

**Fig 3:**
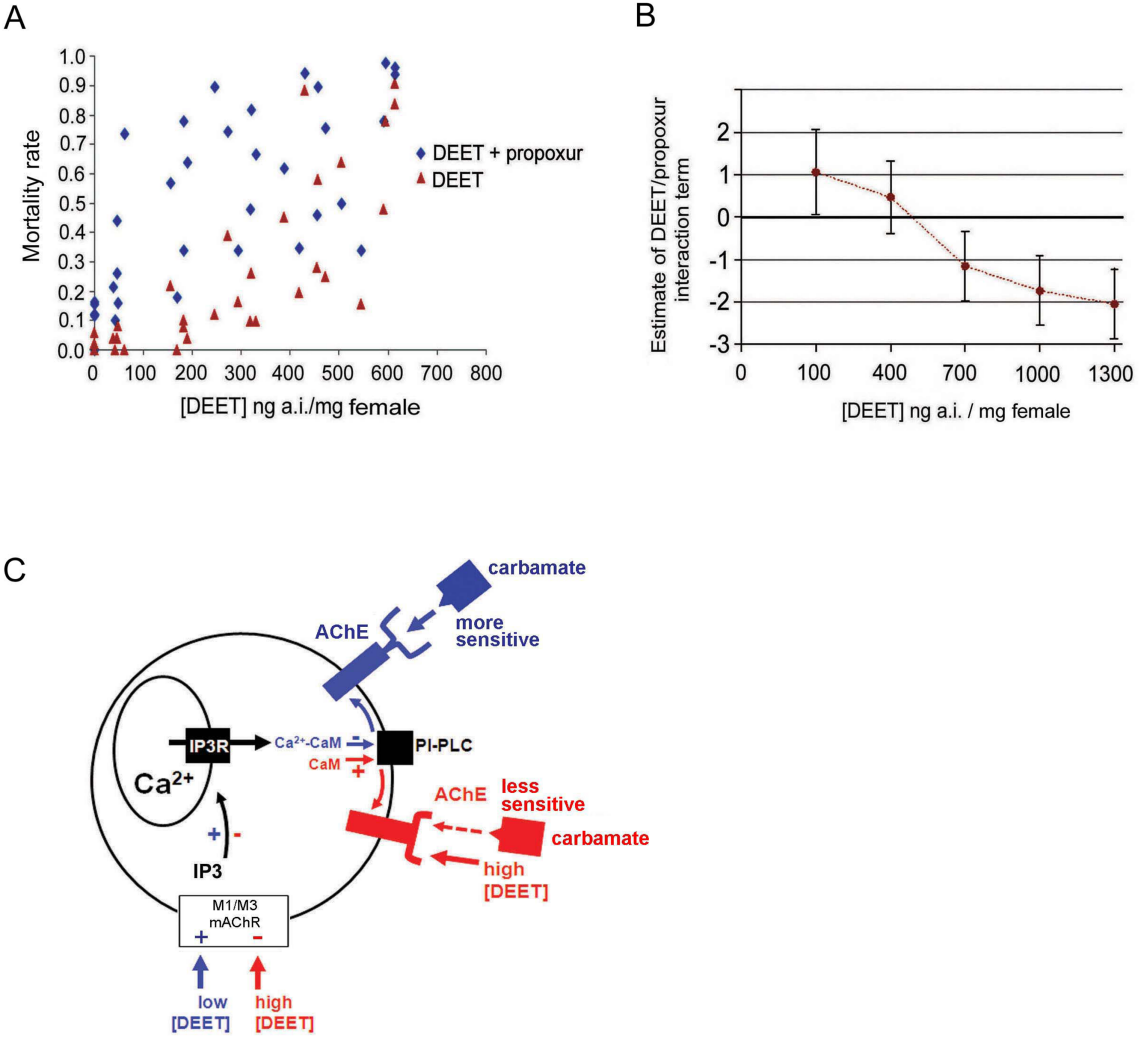
Synergism between DEET and propoxur is effective *in vivo*, in female mosquitoes *Aedes aegypti*. A) Mortality rates relative to the increasing concentrations of DEET (ng of active ingredient (a.i.) / mg female) applied on the thorax of females *Aedes aegypti* in the presence/absence of propoxur (red triangles represent the mortality rates induced by increasing doses of DEET alone and blue diamonds represented the increase of mortality rates when increasing doses of DEET were combined with propoxur at LD_10_). B) Variation of the estimate of DEET/propoxur interaction term in our general linear model relative to the applied doses of DEET. When the interaction term is significantly above 0 (non overlapping of 95%CI), the interaction between DEET and propoxur is synergistic; when the interaction term is significatly below 0, the interaction is antagonistic. C) Proposed model summarizing the essential components of the intracellular signaling pathway that may explain the synergism between DEET and propoxur in insect cell (see text for details). AChE, acetylcholinesterase; CaM, calmodulin; PI-PLC, phosphatidylinositol (PI)-specific phospholipase C; IP3, inositol 1,4,5-triphosphate; IP3R, receptor; mAChR, muscarinic ACh receptor.

To investigate the interaction between DEET and propoxur *in vivo*, we made topical applications of increasing doses of DEET on the thorax of female *Ae*. *aegypti* in the presence/absence of propoxur used at LD_10_ (the lethal dose for 10% of exposed mosquitoes). The mortality rates of *Ae*. *aegypti* relative to the increasing concentrations of DEET applied alone and combined with propoxur at LD_10_ (Fig 3A) were compared. The variation of the estimate of the DEET/propoxur interaction term in our general linear model (S1 Table) relative to the applied doses of DEET (Fig 3B) indicated that when the interaction term was significantly above 0 the interaction between DEET and propoxur was synergistic. By contrast, when the interaction term was significatly below 0, the interaction was considerd as antagonistic. These results, which correlated well with the *in vitro* studies, confirm that interaction between DEET and propoxur switches from antagonism to synergism with low doses of DEET, resulting in a significant increase of the mortality rate of *Ae*. *aegypti*.

### DEET and mammalian mAChRs

**Fig 4:**
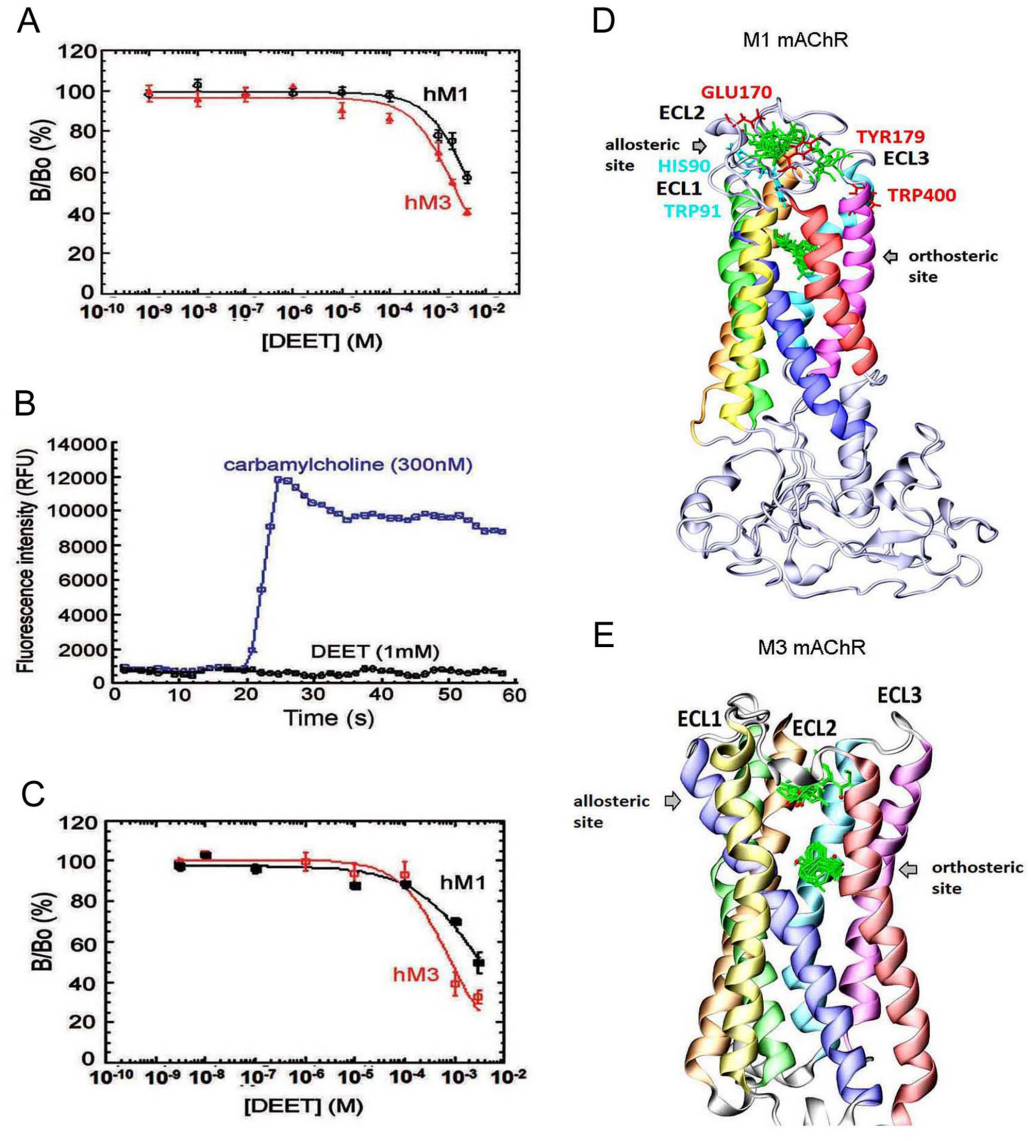
DEET interacts with mammal M1 and M3 muscarinic receptors. The functional properties of DEET are investigated using CHO cells expressing human M1 (hM1) and M3 (hM3) mAChR subtypes. A) Dose-dependent inhibition of [^3^H]-NMS binding to M1 and M3 human muscarinic ACh receptor (mAChR) subtypes by DEET. The results are expressed as the ratio of the specific [^3^H]-NMS binding measured with (B) or without DEET (Bo). B-C) Signals acquired for calcium fluorescence after the addition at 20 sec of carbamylcholine (CCh) (300nM) and DEET (1mM) on CHO-hM1 cells (n = 3). DEET inhibition of the Ca^2+^ mobilization after pretreatment of the cells with increasing concentrations of DEET (3nM to 3 mM), followed by a sub-maximal concentration of CCh (100 nM) (C). D-E) *in silico* Docking of DEET into human M1 and rat M3 mAChRs. The two binding regions (allosteric and orthosteric sites) of DEET molecules (green) in human M1 mAChR are represented (D). In red are shown residues of a M1 mAChR monomer interacting with MT-7 loops previously indentified. Analogous interaction residues from the second M1 mAChR monomer are indicated in blue (D) close to the hypothetical M1 mAChR allosteric site occupied by DEET. The *in silico* docking results to rat M3 mAChR crystal structure 4DAJ (E) also show that DEET binds on two distinct sites (allosteric and orthosteric sites). Ten DEET poses from each group located in both sites are shown in green. The following colour coding for transmembrane helices used: TM1—orange, TM2—green, TM3—dark blue, TM4—yellow, TM5—red, TM6—magenta, TM7—light blue. ECL, extracellular loop.

We then asked whether DEET could affect human M1 and M3 mAChRs. Binding experiments performed on CHO cells stably expressing human M1 and M3 mAChRs showed that DEET displaced the [^3^H]N-Methyl Scopolamine (NMS) binding from M1 and M3 mAChRs but only in the millimolar range. Even if the complete competition binding curves could not be obtained, due to the maximal DMSO percentage tolerated in the assay, the affinity constant of the DEET for the two receptors could be calculated according to the Cheng & Prusoff equation and was equal to 0.69 ± 0.19mM and 0.37 ± 0.02mM for the M1 and M3 mAChRs, respectively (Fig 4A). To better characterize the DEET-mAChR interactions, its effect was also evaluated by measuring changes in [Ca^2+^]i. First, the effect of increasing concentrations of DEET (nM to mM) was evaluated directly on the cells and the signal was compared to control condition obtained with the cholinergic agonist CCh (300nM). As observed (Fig 4B), for the M1 receptor and also observed on M3 receptor (data not shown), addition of relatively high concentration of DEET (1mM) did not induce intracellular calcium release as it is the case for CCh. Thus, DEET, used at high concentration, was not considered as an agonist of both receptor subtypes. Pre-incubation of the cells with increasing concentrations of DEET abolished, in a dose-dependent manner, the CCh-induced activation of both M1 and M3 mAChRs (Fig 4C). The antagonist potency of DEET on both mAChRs was estimated to be 3 ± 1mM and 0.7 ± 0.2mM, in good agreement with the affinity determined in binding experiments. These results indicate that DEET, only displays a low affinity antagonist profile on human M1 and M3 mAChR in a relatively high concentration range. But the most interesting feature is that we have never observed a positive modulation of these mAChRs function at low concentrations of DEET (Fig 4A–4C), suggesting the existence of a selective high affinity positive allosteric site for DEET in insects.

We next carried out experiments by studying the binding site of DEET in human M1 mAChR. Recently, M2 and M3 mAChR structures were published [37,38]. However no crystallographic structure of human M1 mAChR subtype is available yet. MambStruk computational method to predict M1 mAChR structure and the HierDock method were initially used to determine the binding sites of a series of M1 mAChR agonists and antagonists [39]. Recently, it was possible to construct another 3D model of M1 mAChR through the fragmental homology modeling procedure [40]. A model of a dimeric human M1 mAChR, which binds the MT-7 toxin was also developed [41] and very recently, allosteric modulation was studied using homology model of M1 mAChR based on human β2 adrenoreceptor [42].

**Fig 5:**
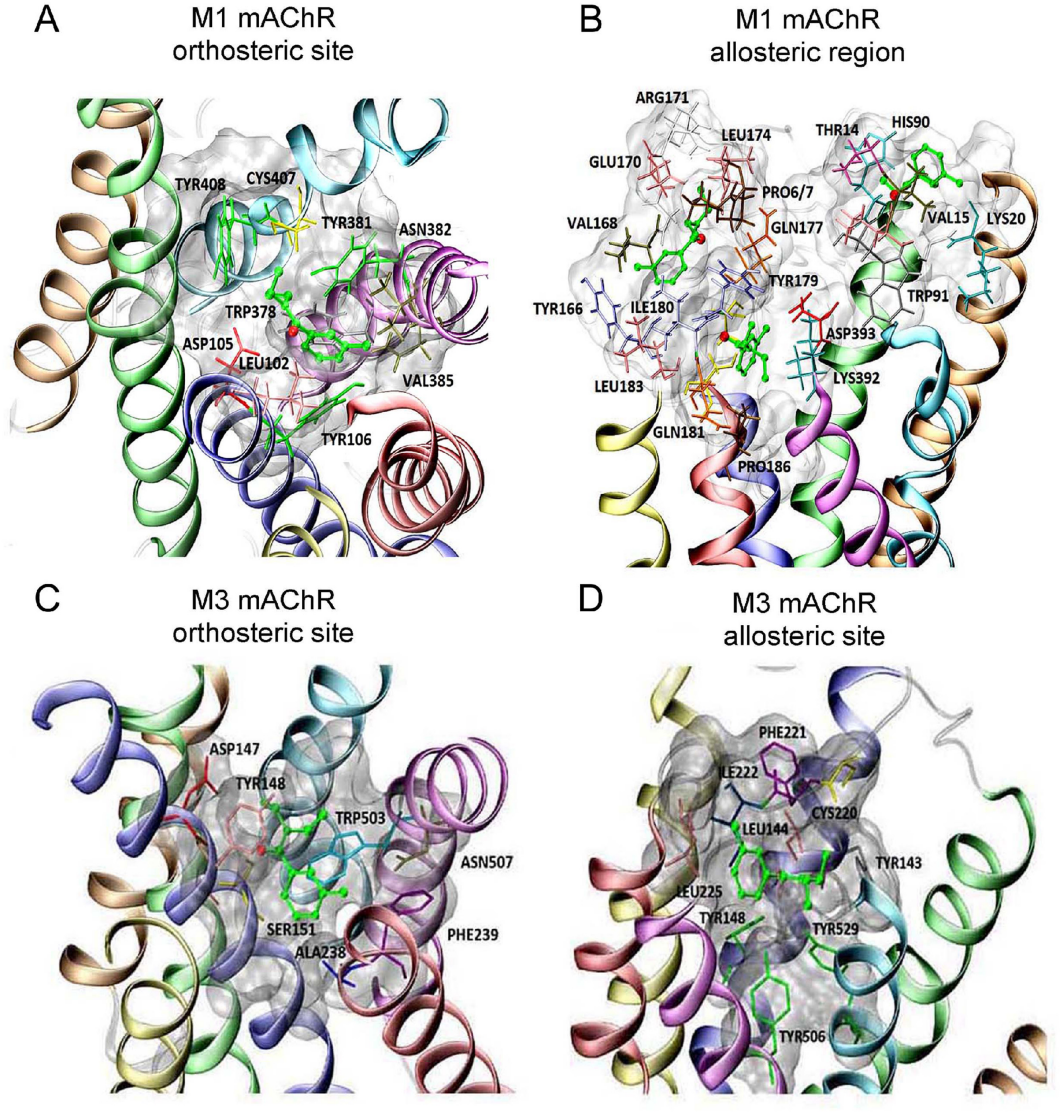
Orthosteric and allosteric binding sites of mammal M1 and M3 muscarinic receptors. In all panels, the M1 and M3 mAChRs orthosteric and allosteric binding sites are shown with the ligand DEET in green. Residues observed in vicinity of DEET in M1 mAChR orthosteric site (A) and allosteric region (B) and in M3 mAChR orthosteric site (C) and allosteric site (D) are shown in licorice representations. The following colour coding for transmembrane helices used: TM1—orange, TM2—green, TM3—dark blue, TM4—yellow, TM5—red, TM6—magenta, TM7—light blue.

Despite different approaches used in modeling strategies, we may conclude that the fold and transmembrane (TM) helices arrangements of all the discussed M1 mAChR structures were similar. Here, the AutoDock 4.2 code was used to determine plausible DEET binding sites in M1 and M3 mACh receptors [43,44]. Because our *in silico* docking of ACh molecule led to the same pose as reported earlier, we performed, for the first time, *in silico* docking of DEET to M1 mAChR model (Fig 4D) using the same methodology. A cluster of poses (from the range #30-#120) was identified in the vicinity of the extracellular loops ECL2 (Figs 4D and 5B) having binding energies (from -4.12 to -3.47 kcal/mol). These poses were grouped into three binding regions located between ends of TM1-TM2, TM4-TM5 and TM5-TM6 helices. Since this area, particularly the TM4-TM5 region, was close to the region identified as the MT-7 neurotoxin site in M1 mAChR [41] outlined by residues GLU170, TYR179, HIS90, and TRP91, we hypothesized that this could be part of high affinity allosteric M1 mAChR site, located extracellularly (Figs 4D and 5B). Alternatively, *in silico* docking place was located on a second site close to the ACh site from the extracellular side. The amino acids building the DEET binding pocket (orthosteric site) were indicated (Figs 4D and 5A). The detailed distances between DEET molecules docked and M1 mAChR residues were presented, (S2 Table).

The *in silico* docking of DEET to the rat M3 crystallographic structure (pdb code 4DAJ) gave similar results (Fig 4E). The majority of DEET poses were located in the orthosteric site (Fig 5C), however, a second cluster of poses was revealed in the vicinity of ECL2, a region which corresponded to the high affinity allosteric site (Figs 4E and 5D, S2 Table). Based on *in silico* docking results with M1 and M3 mAChRs, we expected that, at low concentration, DEET molecules occupied preferentially these allosteric sites. These are in good agreement with *in vitro* studies.

## Conclusion

We report here that mixing low doses of the repellent DEET with the carbamate propoxur increases mortality rate of female *Ae*. *aegypti*. The first attractive aspect of this study is that *in vitro* studies performed in insect neurons, showing synergism between these two compounds, correlate well with the increased lethal effect observed *in vivo*. The molecular events underlying the synergism are summarized as shown (Fig 3C). DEET interacts with positive cooperativity and high affinity on M1/M3 mAChR allosteric site, potentiating the effect of cholinergic agonist on mAChRs. This leads to activation of PLC causing IP3 production. The latter produces the release of calcium from internal stores, which results in the calcium/CaM complex formation reducing the PI-PLC activity involved in the regulation of AChE. This finally renders AChE more sensitive to propoxur. At the opposite, high concentrations of DEET binds with low affinity on distinct mAChR interaction site inducing antagonism function, which decreases AChE sensitivity to propoxur.

Previous data have reported synergism between DEET and propoxur in *Aedes aegypti* [18]. Because this synergistic interaction disappears in the presence of piperonyl butoxide (PBO), the involvement of cytochrome-P450 monooxygenases is supposed to be essential for this positive interaction observed between DEET and propoxur [18]. However, even if the precise molecular event involved in the synergism is still unknown, the results reported in the present study may help to understand further the physiological implication of cytochrome-P450 monooxygenases in the synergistic interactions occurring between DEET and propoxur. Previously, it has been shown that intracellular calcium ions may mediate calcium-dependent up-regulation of cytochrome-P450 monooxygenases via the activation of calcium/calmodulin-dependent protein kinase [45,46]. Based on these ensemble outcomes, it is tempting to postulate that DEET-induced intracellular calcium rise occurring, via the mechanism described above, may also serve as an additional calcium-dependent effector mechanism to regulate basal activity of cytochrome-P450 monooxygenases, which will thereby increase the synergism between DEET and propoxur.

Finally, the second interesting aspect of this present study is that i*n vitro* studies and *in silico* docking performed on human M1/M3 mAChRs indicate that the positive interaction does not exist, revealing a new insect selective site. These findings open novel perspectives to maximize prevention against insects that transmit many deadliest diseases. The increased efficiency, based on the positive interaction between two compounds will help to design more adapted treatments to control insect vector-borne diseases.

## Materials and Methods

### Cell isolation, whole-cell patch-clamp technique and statistical analysis

Experiments were carried out on insect neurosecretory cells identified as Dorsal Unpaired Median (DUM) neurons [19,20] isolated from the midline of the terminal abdominal ganglion (TAG) of the nerve chord of adult male cockroaches (*Periplaneta americana)*. Cockroaches were obtained from our laboratory stock colonies maintained at 29°C on 12h light/dark cycle. Animals were immobilized ventral side up on a dissection dishes. The ventral cuticle and the accessory gland were removed to allow access to the TAG which was carefully dissected under a binocular microscope and placed in normal cockroach saline containing (in mM): 200 NaCl, 3.1 KCl, 5 CaCl_2_, 4 MgCl_2_, 10 HEPES, 50 sucrose and pH was adjusted to 7.4 with NaOH. Isolation of adult DUM neuron somata was performed under sterile conditions using enzymatic digestion by collagenase (Type IA, 300 IU/mL; Worthington Biochemicals, Lakewood, NJ, USA) at 29°C during 35 minutes. Then, a mechanical dissociation through fire-polished Pasteur pipettes was used in order to isolate DUM neurons from the TAG [47]. DUM neuron somata were maintained at 29°C for 24h before electrophysiological experiments were carried out.

ACh- and muscarine-induced currents were recorded using the patch-clamp technique in the whole-cell recording configuration under voltage-clamp mode. Ejection pipettes and patch-clamp electrodes were pulled from borosilicate glass capillary tubes (GC150T-10; Clark Electromedical Instruments Harvard Apparatus, UK) using a P-97 model puller (Sutter Instruments, USA). Patch pipettes had resistances ranging from 1 to 1.2MΩ when filled with internal pipette solution (see composition below). The liquid junction potential between extracellular and intracellular solutions was always corrected before the formation of a gigaohm seal (>3GΩ). Signals were recorded with an Axopatch 200A (Axon instruments, USA). Ionic currents induced by ACh were displayed on a computer with software control pClamp (version 6.0.3, Axon Instruments, USA) connected to a 125-kHz labmaster DMA data acquisition system (TL-1; Axon Instruments, USA). DUM neuron somata were voltage-clamped at a steady-state holding potential of -50mV (except when otherwise stated). Experiments were carried out at room temperature. Bath solution superfusing the cells contained (in mM): 200 NaCl; 3.1 KCl; 5 CaCl_2_; 4 MgCl_2_; 10 HEPES; pH was adjusted to 7.4 with NaOH. Patch pipettes were filled with solution containing (in mM): 160 K^+^/D-Gluconate; 10 KF; 10 NaCl; 1 MgCl_2_; 0.5 CaCl_2_; 3 ATP; 0.1 cAMP; 10 EGTA; 20 HEPES; pH was adjusted to 7.4 with KOH.

ACh (1M) and muscarine (10mM) were applied by pneumatic pressure ejection (15psig) [22,23,28,29] with a pneumatic pressure system (Miniframe, Medical System Corporation, USA) to minimize receptor desensitization resulting from bath application of agonists. The pressure ejection was made through a controlled calibrated patch pipette geometry obtained according to the protocol described just above (with a resistance of 1.8MΩ when filled with agonists) positioned in extracellular solution within 50μm from the isolated neuron cell body. Using this protocol, the logarithmic concentration of cholinergic agonist, at any point of the cell body, will be proportional to the pulse duration of the cholinergic agonist applications (at constant pressure), as previously reported on the same preparation^16^. Pharmacological agents such as α-bungarotoxin (α-bgt; 0.5μM), DEET (used at different concentrations ranging from 10nM to 1μM), caffeine (10mM), TBM-8 (100μM), BW284c51 (100nM), pirenzepine (100nM), 4-DAMP (100nM), MT-7 (30nM), U73122 (10μM), *m*-3M3FBS (10μM) and propoxur (100nM) were added to external solution. W7 (0.5mM), BAPTA (10mM), KN-62 (10μM) and calmodulin (50nM) were added in the internal pipette solution immediately before use.

Data analysis was performed using the software Prism 5 (Graph Pad Software, San Diego, CA). Data were analysed using one-way ANOVA and data are presented as mean ± S.E.M (Standard Error Mean).

### Calcium imaging

Falcon 1006 Petri dishes with glass coverslips were coated with poly-D-lysine hydrobromide (mol. wt. 70,000–150,000; Sigma Chemical, l’Isle d’Abeau Chesnes, France), and isolated DUM neuron cell bodies were plated as described above. External recording solution contained (in mM): 200 NaCl; 3.1 KCl; 5 CaCl_2_; 4 MgCl_2_, and 10 HEPES buffer; pH was adjusted to 7.4 with NaOH. The cells were incubated in the dark with 10μM Fura-2 pentakis (acetoxy-methyl) ester (Sigma Chemical) for 60min at 37°C. After loading, cells were washed three times in saline. The glass coverslips were then mounted in a recording chamber (Warner Instruments, Hamden, CT) connected to a gravity perfusion system allowing drug application. Imaging experiments were performed with an Olympus IX50 inverted microscope (Olympus, Rungis, France) equipped with epifluorescence. Excitation light was provided by a 75-W integral xenon lamp (Life Science Resources, Cambridge, UK). Excitation wavelengths (340nm and 380nm) were applied using a computer-driven Spectramaster (Life Science Resources). Images were collected with an Olympix digital charge-coupled device (CCD) camera (AstroCam; Life Science Resources), and they were recorded in the computer with Merlin software, version 2.0 (Life Science Resources). Exposure times at 340nm and 380nm were usually 150ms, and images were collected at various frequencies. Data were expressed as the ratio of emitted fluorescence (340/380nm) [48].

### Biochemical assay of acetylcholinesterase activity

Six TAG of the ventral nerve cord of adult male cockroach *Periplaneta americana* were placed in 1200μL normal cockroach saline buffer containing (in mM): 200 NaCl; 3.1 KCl; 5 CaCl_2_; 4 MgCl_2_; 50 sucrose and 10 HEPES, pH was adjusted to 7.4 with NaOH. Isolation of DUM neuron cell bodies was performed as previously described just above. 90μL of the suspension were incubated for 15min with 10μL of the carbamate insecticide, propoxur. Dilutions (10^-5^M, 10^-6^M, 5.10^-7^M, 10^-8^M and 10^-9^M) from the initial concentration (1M) were used for propoxur. Acetylcholinesterase (AChE) residual activity was determined spectrophotometrically according to the method described elsewhere [49], at 405 nm at 30°C for 30min using 100μL of 0.1mM acetylthiocholine (ATC) (Sigma-Aldrich, St Quentin-Fallavier, France) and 100μL of 1mM DTNB (5,5'-dithiobis-(2-nitrobenzoic acid), Sigma-Aldrich) for each dilution, and was expressed as a percentage of initial activity (i.e., without insecticide).

### Synaptic transmission—Single-fibre oil-gap method

The TAG with the nerve cord were carefully dissected and placed in normal cockroach saline containing (in mM): 208 NaCl; 3.1 KCl; 10 CaCl_2_; 26 sucrose; 10 HEPES; pH was adjusted to 7.2 with NaOH. The synaptic preparation was composed of a cercus, the corresponding cercal nerve XI, the de-sheathed TAG (containing the studied synapse) and the abdominal part of the nerve cord. Electrophysiological recordings of synaptic events were obtained using the single-fibre oil-gap method [36]. With this technique, it is possible to record the cholinergic synaptic transmission by measuring the post-synaptic polarisation. During experiments, this resting potential was continuously monitored on a pen chart recorder. DEET (used at various concentrations) and atropine (1μM) were bath-applied directly onto the TAG during periods of 30–60min. Experiments were conducted at room temperature (20°C). Data were expressed as a mean ± S.E.M..

### Binding of [^3^H]N-Methylscopolamine assays

CHO cells stably expressing the human M1 and M3 muscarinic receptors (kindly provided by Prof. P. O. Couraud; ICGM, Paris, France) were grown in plastic Petri dishes incubated at 37°C in an atmosphere of 5% CO_2_ and 95% humidified air in Ham F12 medium pre-complemented with L-glutamine and bicarbonate (Sigma-Aldrich), supplemented with 10% fetal calf serum and 1% penicillin/streptomycin (Sigma-Aldrich). At 100% confluence, the medium was removed and the cells were harvested using a Versene buffer (PBS+5mM EDTA). The cells were washed with ice-cold phosphate buffer and centrifuged at 1700g for 10min (4°C). The pellet was suspended in ice-cold buffer (1mM EDTA, 25mM Na phosphate, 5mM MgCl_2_, pH 7.4) and homogenized using an Elvhjem-Potter homogenizer (Fisher Scientific Labosi, Elancourt, France). The homogenate was centrifuged at 1700g for 15min (4°C). The sediment was re-suspended in buffer, homogenized and centrifuged at 1700g for 15min (4°C). The combined supernatants were centrifuged at 35000g for 30min (4°C) and the pellet re-suspended in the same buffer (0.1mL/dish). The membrane preparations were aliquoted and stored at -80°C. Protein concentrations were determined by the Lowry method using bovine serum albumin as standard.

All binding experiments of [^3^H]N-Methylscopolamine ([^3^H]N-MS) were carried out at room temperature, in 10mM sodium phosphate, pH 7.2, 135mM NaCl, 2.5mM KCl pH 7.4, 0.1% bovine serum albumin (PBS-BSA). The effect of DEET on the equilibrium binding of a fixed concentration of [^3^H]N-MS was determined in inhibition experiments. CHO-hM1 and CHO-hM3 membranes, at a protein concentration where no more than 10% of added radioligand was bound (ca. 1500cpm), were incubated overnight in PBS-BSA at 25°C, with [^3^H]NMS (0.5nM) and varying concentrations of DEET, in a final assay volume of 300μL. Nonspecific binding was determined in the presence of 50μM atropine. The reaction was stopped by addition of 3mL of ice-cold buffer (Tris 10mM), immediately followed by filtration through Whatman GF/C glass fibre filters pre-soaked in 0.5% polyethylenimine. The filters were washed once with 3mL ice-cold buffer (PBS), dried, and the bound radioactivity counted by liquid scintillation spectrometry. Each experiment was done at least three times. The binding data from individual experiments (n = 3) were analyzed by nonlinear regression analysis using Kaleidagraph 4.0 (Synergy Software, Reading, PA). The affinities of DEET in inhibiting the binding of [^3^H]N-MS, expressed as K_i,_, were calculated from the IC_50_ values by applying the Cheng-Prussoff correction [Ki = IC_50_/(1 + L*/Kd)], with Kd NMS for human M1 and M3 equal to 0.1nM. [^3^H]N-MS, (78Ci/mmol) was from PerkinElmer Life Sciences (Courtaboeuf, France). Carbamylcholine and atropine were from Sigma-Aldrich.

### Functional calcium assays

CHO cells stably expressing human M1 or M3 receptors were plated (30000 to 50000 cells/well in 100μL) on black-walled 96-well plates (Greiner). 24h after coating, the cells were first incubated with DEET for 45min and then for 45 additional min with the CaKit dye resuspended in Hank Balanced Salt Solution (HBSS) buffer complemented with HEPES 20mM, at pH 7.4 (R8041, Molecular Devices Ltd, Wokingham, UK). The fluorescence was recorded using a FLEXstation II plate reader (Molecular Devices Ltd) with excitation and emission wavelengths fixed at 485nm and 525nm, respectively. Drug dilutions in assay buffer were prepared in a separate 96-well plate. Parameters for drug addition to the cell plate were preprogrammed and delivery of the agonist carbamylcholine (100nM) was automated through an 8-tip head pipettor, 20sec after the beginning of the recording. Dose-response curves were constructed by measuring the fluorescence intensity after normalization to the maximal response to carbamylcholine, measured in the absence of DEET. All data points were measured in duplicate. FLEXstation calcium assay kit (Molecular Devices Ltd) was the calcium-specific fluorescent dye used in this study and the calcium flux measurement was done on a FLEXstation machine (Molecular Devices Ltd).

### Molecular *in silico* docking of DEET into human muscarinic acetylcholine receptor

The *in silico* docking of DEET into human Muscarinic acetylcholine receptor M1 (M1 mAChR, P11229 UniProtKB) was performed using the well established AutoDock program (version 4.2) [40]. The structure of the receptor was kindly provided by Dr M. Michael Espinoza-Fonesca [34]. The docked ligand was flexible, the protein was frozen, nonpolar hydrogens were united with carbons and charges were determined using the Gasteiger method. For DEET M1-mAChR system 256 starts of the Lamarckian genetic algorithm were initiated with a maximum number of 2,500,000 energy evaluations and a maximum number of 27,000 generations allowed. Grid maps of increasing density were used to determine precisely the optimum DEET *in silico* docking place: 70x70x112Å with grid point spacing 1.0Å or 126 x 126 x 126Å with grid points spacings 0.408Å and 0.375Å. The same protocol was used to prepare DEET *in silico* docking to the rat M3 mAChR crystallographic structure (pdb code ‘DAJ [32]). Analysis of results and figures were prepared using the VMD 1.9 code [41] and home-made scripts.

### Insecticide/repellent interaction studies on mosquitoes

A susceptible strain of *Aedes aegypti* named Bora originating from French Polynesia, which has been colonized in the laboratory for many years and free of any detectable resistance mechanisms was used. Bioassays were carried out with technical grades of active ingredients diluted in acetone. Propoxur (2-isopropoxyphenylmethylcarbamate) 99.6% was provided by Bayer CropScience (Monheim, Germany). DEET 97% was provided by Sigma-Aldrich.

Topical applications were used to measure the interactions occurring between technical insecticide and repellent on *Ae*. *aegypti*. This method allows estimating the intrinsic toxicity of a product excluding all other effects linked to mosquito's behaviour, especially when exposed to an irritating or repellent compound. Non blood-fed females of *Ae*. *aegypti*, aged 2–5 days, were first anaesthetised by limited contact with carbon dioxide (45 sec) and deposited on a cold plate (4°C) to maintain anaesthesia during manipulation. Fifty females were used for each dose. A volume of 0.1μL of acetone solution (containing the product(s) at the required concentration(s)) was applied on the upper part of female's pronotum using a micro-capillary. Fifty females that received 0.1μL of pure acetone served as control. Females were preserved at 4°C on the cold plate during this interval of time, to ensure the diffusion of enzyme inhibitor through mosquito body prior to insecticide treatment. After manipulation, females were transferred into plastic cups, provided with sugar solution and held for 24 hours at 27°C and 80% RH. Mortality rates were recorded 24 hours after testing. Data were expressed in nanograms of active ingredient per milligram of mosquito female body weight. Six replicates were done for each tested concentration using different batches and generations of mosquitoes.

Data of mortality induced by DEET alone and in combination with propoxur were analyzed through a logistic regression using the proportion of dead mosquitoes among the mosquitoes exposed to chemicals as the response variable. The analysis consisted in logistic regression modeling of the probability of a mosquito to die in relation to explanatory variables:

- concentration of DEET,
- mean weight of tested mosquitoes (for each replicate),
- presence of a dose of propoxur killing 10% of exposed mosquitoes when used alone,
- interaction between DEET and propoxur

Using these explanatory variables, we performed an analysis of deviance in order to construct the final general linear model. All the analyses were performed using R solftware [17].

## Supporting Information

S1 Fig Isolated DUM neuron cell bodies express functional acetylcholinesterase (AChE). A) Semi-logarithmic dose-response curve for the acetylcholine-induced depolarization shown *in inset*. Acetylcholine (1M, 15psig, 300ms, corresponding to the ED_50_, which is the Effective pressure ejection Duration of ACh needed to obtain half of the maximal response and to avoid receptor desensitization) is applied by pneumatic pressure ejection onto isolated DUM neuron cell bodies held at a holding potential of -48mV. The smooth line represents the best fit (r = 0.998) through the mean data points according to the Hill equation. B-C) Comparative histograms showing the anticholinesterase effect of DEET (1μM) and propoxur (100nM), applied alone, on the duration of ACh-induced currents. Interestingly, when DEET and propoxur were applied in combination on the isolated DUM neuron cell bodies, pretreated by either DEET (1μM) or propoxur (100nM) for 10 minutes, no additional effects were observed in both experimental conditions. This indicates that DEET and propoxur act similarly on the same target (i. e., AChE). Data are means ± S.E.M. (n = 10 to 16 cells; Ctr: control, ns: not significant, ** values significantly different *p* < 0.01).
(EPS)

S2 Fig Selective M1/M3 muscarinic acetylcholine receptor subtype antagonists and intracellular calcium affect the anticholinesterase effects of DEET and propoxur and the synergism between DEET and propoxur. A-B) Bath application of the selective M1/M3 mAChR subtypes antagonists, pirenzepine (PZP) and 4-DAMP reduce the anticholinesterase effect of DEET (10nM) (A). By contrast, these antagonists do not produce any significant effects on propoxur-induced anticholinesterase action (B). C) Histogram summarizing that high calcium buffering using intracellular perfusion of 10mM BAPTA reduces the effect of propoxur observed on the duration of the ACh-induced currents. Opposite effect is obtained when DUM neuron cell body is treated with caffeine, known to stimulate the release of calcium from internal stores. Caffeine (10mM) induced a strong potentiation of the anticholinesterase effect of propoxur, as it is observed with DEET (10nM). This indicates the existence of an intracellular calcium-dependent mechanism involved in the synergism between DEET and propoxur. D) Even in the presence of PZP, it is possible to counteract the inhibitory effect of these antagonists on the synergism between DEET and propoxur by increasing internal calcium concentration (i.e., without EGTA in the patch pipette). Number of experiments varies from 8 to 14 cells. Data are means ± S.E.M. (Ctr: control, ns: not significant, ** and ***, values significantly different *p* < 0.01 and *p* < 0.001, respectively).
(EPS)

S3 Fig Dose-dependent opposite effects of DEET on insect synaptic muscarinic ACh receptors. A) Scheme illustrating the perspex experimental chamber suitable for studying the cholinergic synaptic transmission using the single-fiber oil-gap technique [36]. The cockroach *Periplaneta americana* synaptic preparation is composed of a cercus, the corresponding cercal nerve XI, the de-sheathed Terminal Abdominal Ganglion (TAG), containing the studied synapse and the abdominal part of the nerve cord. With this electrophysiological technique it is possible to record the effect of DEET resulting from its interaction with post-synaptic muscarinic ACh receptors (mAChR) of giant interneuron (GI). B) Typical example of unitary excitatory postsynaptic potentials (uEPSP) reflecting spontaneous activity of presynaptic cercal mechanoreceptors. C) Bath application of 500nM DEET produces a depolarization of the postsynaptic membrane, which is completely inhibited by the mAChR antagonist atropine (1μM). By contrast, higher concentration of DEET (10μM) fails to induce any significant variation of the postsynaptic potential. D) Comparative histogram illustrating the unexpected dose-dependent effect of DEET on the post-synaptic mAChRs. Low concentrations of DEET (100nM and 500nM) increase the postsynaptic membrane depolarization amplitude. By contrast, higher concentration of DEET (10μM) produces an opposite effect. Data are means ± S.E.M. (n = 4). St, stimulation; TAG, Terminal Abdominal Ganglion, Cs, cercus; A, amplifier; and recording system; b, c, saline compartments; d, oil compartment.
(EPS)

S1 Table Summary of the generalized linear model of mortality. Through topical applications on the mosquito thorax of active ingredients in an ethanol solution, we investigated the dose-dependent relationship between DEET and a single dose of propoxur (raw data are illustrated in the Fig 3A). Response variable was the mortality and the explanatory variable were the DEET concentration, the mean mass of the mosquito tested, and the addition of the propoxur (prop). A logit link and binomial error structure were used and interactions were defined as products. Coefficients were given along with their standard errors. Treatment contrasts were used and the significance of the main effects and interaction terms was < 0.05. The residual deviance was 4458 on 4904 r.d.. The weight of the mosquitoes explained a significant part of the deviance, leading us not to remove this term of the model. The dose-dependent pattern of the interaction term, illustrated in the Fig 3B, indicates that the interaction between DEET and propoxur switches from synergism to antogonism with increasing concentrations of DEET (s.e., standard error).
(DOC)

S2 Table Amino acid residues involved in the interactions of DEET with the different sites of mAChR subtypes. Residues located in a close proximity to DEET docked poses found in the allosteric regions and in the orthosteric site of human M1 mAChR model (Figs 4D, 5A and 5B). Residues involved in the interactions with MT-7 toxin, i.e. members of the allosteric site determined experimentally, are shown in bold. Residues interacting with MT-7 and present in M1 ECL2 loop are shown in italics. In the second part of the table, residues located in the close proximity of the poses of DEET found in allosteric and orthosteric sites of rat M3 mAChR receptor are listed as well (Figs 4E, 5C and 5D). mAChR, muscarinic acetylcholine receptor; TM, transmembrane; Ang., Angström.
(DOC)
