# Supplementary material for: The Repellent DEET Potentiates Carbamate Effects via Insect Muscarinic Receptor Interactions: An Alternative Strategy to Control Insect Vector-Borne Diseases
Source: PLoS One. 2015 May 11;10(5):e0126406. doi: 10.1371/journal.pone.0126406 (PMC4427492; doi:10.1371/journal.pone.0126406)
Supplement: S1 Table — Through topical applications on the mosquito thorax of active ingredients in an ethanol solution, we investigated the dose-dependent relationship between DEET and a single dose of propoxur (raw data are illustrated in the Fig 3A). Response variable was the mortality and the explanatory variable were the DEET concentration, the mean mass of the mosquito tested, and the addition of the propoxur (prop). A logit link and binomial error structure were used and interactions were defined as products. Coefficients were given along with their standard errors. Treatment contrasts were used and the significance of the main effects and interaction terms was < 0.05. The residual deviance was 4458 on 4904 r.d.. The weight of the mosquitoes explained a significant part of the deviance, leading us not to remove this term of the model. The dose-dependent pattern of the interaction term, illustrated in the Fig 3B, indicates that the interaction between DEET and propoxur switches from synergism to antogonism with increasing concentrations of DEET (s.e., standard error). (DOC) [file pone.0126406.s004.doc]

**Supplementary information Table S1 Summary of the generalized linear model of mortality.**

| Main effect terms | Estimates | s.e. | p-value |
| --- | --- | --- | --- |
| intercept ([DEET]=0, without propoxur, mean weight=1,63mg) | -2.7751 | 0.3679 | 4.54e-14 |
| propoxur (LD_10_) | 2.5031 | 0.3853 | 8.17e-11 |
| deet1 | 0.2786 | 0.4837 | 0.56465 |
| deet2 | 1.6345 | 0.4032 | 5.02e-05 |
| deet3 | 3.6253 | 0.3772 | < 2e-16 |
| deet4 | 4.3444 | 0.3772 | < 2e-16 |
| deet5 | 5.2212 | 0.3806 | < 2e-16 |
| mean weight=1.88mg | -1.0622 | 0.1554 | 8.28e-12 |
| mean weight=2.12mg | -1.6985 | 0.1549 | < 2e-16 |
| mean weight=2.2mg | -2.2951 | 0.1583 | < 2e-16 |
| mean weight=2.31mg | -0.9856 | 0.1525 | 1.02e-10 |
| mean weight=2.33mg | -1.1314 | 0.155 | 2.94e-13 |
| mean weight=2.39mg | -3.2825 | 0.1729 | < 2e-16 |
| mean weight=2.58mg | -1.6765 | 0.1526 | < 2e-16 |
| Interaction terms |  |  |  |
| prop1:deet1 | 1.0629 | 0.5142 | 0.03872 |
| prop1:deet2 | 0.4722 | 0.4399 | 0.28305 |
| prop1:deet3 | -1.153 | 0.4158 | 0.00554 |
| prop1:deet4 | -1.7354 | 0.4162 | 3.04e-05 |
| prop1:deet5 | -2.0454 | 0.4212 | 1.19e-06 |
