# Supplementary material for: The Repellent DEET Potentiates Carbamate Effects via Insect Muscarinic Receptor Interactions: An Alternative Strategy to Control Insect Vector-Borne Diseases
Source: PLoS One. 2015 May 11;10(5):e0126406. doi: 10.1371/journal.pone.0126406 (PMC4427492; doi:10.1371/journal.pone.0126406)
Supplement: S2 Table — Residues located in a close proximity to DEET docked poses found in the allosteric regions and in the orthosteric site of human M1 mAChR model (Figs 4D, 5A and 5B). Residues involved in the interactions with MT-7 toxin, i.e. members of the allosteric site determined experimentally, are shown in bold. Residues interacting with MT-7 and present in M1 ECL2 loop are shown in italics. In the second part of the table, residues located in the close proximity of the poses of DEET found in allosteric and orthosteric sites of rat M3 mAChR receptor are listed as well (Figs 4E, 5C and 5D). mAChR, muscarinic acetylcholine receptor; TM, transmembrane; Ang., Angström. (DOC) [file pone.0126406.s005.doc]

**Supplementary information Table S2 Amino acid residues involved in the interactions of DEET with the different sites of mAChR subtypes.**

| **Human M1 mAChR** | | **Range** | | | | |  |
| --- | --- | --- | --- | --- | --- | --- | --- |
|  |  | **< 3 Ang.** | | **< 4 Ang.** | **< 5 Ang.** | |  |
| **Allosteric**  **regions** | |  | |  |  | |  |
| TM1-TM2 | | THR14, LEU16, GLY19, PRO22, VAL 25, **HIS90**, **TRP91**, ALA92 | | PRO11, ASN12, ILE13, VAL15 | LYS20, GLY21, GLY89 | |  |
| TM4-TM5 | | PRO7, VAL168, GLY169, *GLU170*, *LEU174*, ***TYR179***, ILE180 | | PRO6, TYR166, LEU167, *ARG171*, GLN177, LEU183 | ALA175 | |  |
| TM5-TM6 | | ***TYR179***, ILE180, GLN181, PRO186, LYS392 | | CYS178, PHE390, ASP393 | ASN2, THR3, SER4, GLN185, THR389, CYS394 | |  |
|  | |  | |  |  | |  |
| **Orthosteric**  **site** | | TYR106, TRP378,  TYR381, ASN382,  CYS407 | | CYS98, LEU102,  GLU176, VAL385,  TYR404, TYR408 | ILE74, TYR82, TRP101,SER109, GLN185,THR189, ALA193, PHE197 | |  |
|  | |  | |  |  | |  |
| **Rat M3 mAChR** |  | | **Range** | | |  | |
|  | **< 3 Ang.** | | **< 3.5 Ang.** | | | **< 4 Ang.** | |
| **Allosteric site** | TYR148 | | LEU225, TYR529, LEU144, ILE222 | | | TYR506, TRP143, PHE221, SER226 | |
| **Orthosteric site** | SER151, TYR148 | | ASP147, ALA238 | | | TRP503, ASN507, ASN152, CYS532 | |
